# Improving the Performance of Outcome Prediction for Inpatients With Acute Myocardial Infarction Based on Embedding Representation Learned From Electronic Medical Records: Development and Validation Study

**DOI:** 10.2196/37486

**Published:** 2022-08-03

**Authors:** Yanqun Huang, Zhimin Zheng, Moxuan Ma, Xin Xin, Honglei Liu, Xiaolu Fei, Lan Wei, Hui Chen

**Affiliations:** 1 School of Biomedical Engineering Capital Medical University Beijing China; 2 Beijing Key Laboratory of Fundamental Research on Biomechanics in Clinical Application Capital Medical University Beijing China; 3 Information Center Xuanwu Hospital Capital Medical University Beijing China

**Keywords:** representation learning, skip-gram, feature association strengths, feature importance, mortality risk prediction, acute myocardial infarction

## Abstract

**Background:**

The widespread secondary use of electronic medical records (EMRs) promotes health care quality improvement. Representation learning that can automatically extract hidden information from EMR data has gained increasing attention.

**Objective:**

We aimed to propose a patient representation with more feature associations and task-specific feature importance to improve the outcome prediction performance for inpatients with acute myocardial infarction (AMI).

**Methods:**

Medical concepts, including patients’ age, gender, disease diagnoses, laboratory tests, structured radiological features, procedures, and medications, were first embedded into real-value vectors using the improved skip-gram algorithm, where concepts in the context windows were selected by feature association strengths measured by association rule confidence. Then, each patient was represented as the sum of the feature embeddings weighted by the task-specific feature importance, which was applied to facilitate predictive model prediction from global and local perspectives. We finally applied the proposed patient representation into mortality risk prediction for 3010 and 1671 AMI inpatients from a public data set and a private data set, respectively, and compared it with several reference representation methods in terms of the area under the receiver operating characteristic curve (AUROC), area under the precision-recall curve (AUPRC), and F1-score.

**Results:**

Compared with the reference methods, the proposed embedding-based representation showed consistently superior predictive performance on the 2 data sets, achieving mean AUROCs of 0.878 and 0.973, AUPRCs of 0.220 and 0.505, and F1-scores of 0.376 and 0.674 for the public and private data sets, respectively, while the greatest AUROCs, AUPRCs, and F1-scores among the reference methods were 0.847 and 0.939, 0.196 and 0.283, and 0.344 and 0.361 for the public and private data sets, respectively. Feature importance integrated in patient representation reflected features that were also critical in prediction tasks and clinical practice.

**Conclusions:**

The introduction of feature associations and feature importance facilitated an effective patient representation and contributed to prediction performance improvement and model interpretation.

## Introduction

Electronic medical records (EMRs) contain diverse and heterogeneous information, such as demographic data, disease diagnoses, laboratory tests, radiological findings, examinations and procedures, and medications. EMR data can be used to not only reflect the health status of patients and record the treatment trajectory, but also help doctors in making clinical decisions [1-6] and improving the efficiency of diagnosis and treatment [1,7,8]. One of the most prevalent and practical tasks of the secondary use of EMR data is building models to predict the disease status [8-10] and treatment outcomes [11-17] for a patient, using machine learning algorithms.

However, the high dimensionality, sparsity, and heterogeneity of EMR data [12,18] pose many obstacles for directly inputting the raw data into machine learning–based predictive models. Some manual and data-driven feature engineering methods [15,19], though time-consuming and laborious, were used to select important features or extract useful information for predictive tasks. Moreover, the performance of predictive models relies heavily on the representation of data. It was reported that effective representation methods could make the downstream modeling simpler and more flexible, and greatly improve the predictive performance [18,20]. By transforming raw features into compact vectors, representation learning can make it easier to automatically extract useful information when building predictive models [16,21,22]. One widely used representation method for EMR data is the skip-gram algorithm [23], a distributed embedding method that treats patient records as sentences and medical concepts as words. An inevitable problem in the skip-gram algorithm is that contrary to words within a sentence, medical concepts in a patient’s record do not have a natural order, making it difficult to learn meaningful representations of concepts that have potential associations. One solution for this problem was randomly shuffling the concepts within a record to learn concept embeddings [12,24-26]. It could reduce the impact of the disorder attribute of medical concepts on the algorithm to some degree, while associations among these concepts were still not taken into consideration.

Acute myocardial infarction (AMI) is an acute ischemic heart disease and is the second leading cause of death. One in every 6 deaths is caused by ischemic heart disease, where AMI accounts for the majority of deaths [27,28]. Mortality risk prediction for AMI patients plays a crucial role in clinical work, helping doctors identify potential clinical factors, take early intervention measures based on timely alerts of patients’ adverse health statuses, and reduce the burdensome expenditure of related health care expenses. Therefore, researchers [19,29-31] have focused on building machine learning models for the outcome prediction of AMI patients, and most of them used specific clinical features, such as laboratory test results (eg, albumin), comorbidities (eg, diabetes), and demographic data (eg, gender).

**Figure 1:**
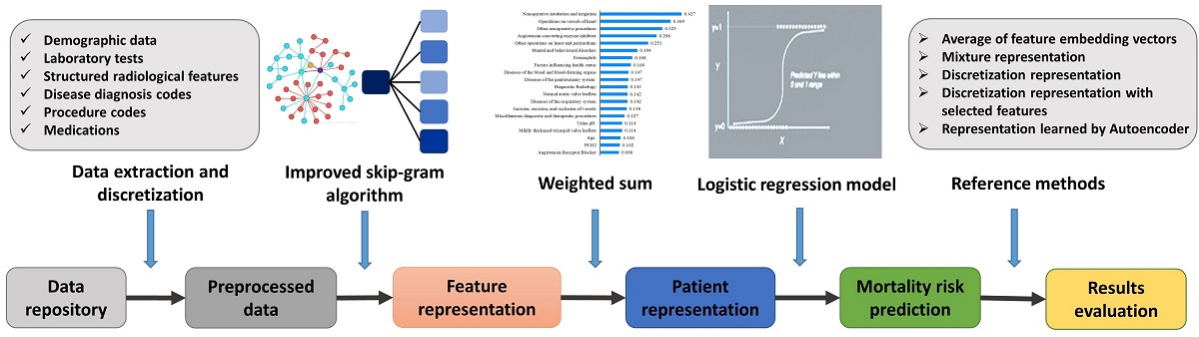
Overview of the proposed representation learning method for patients’ mortality risk prediction. First, feature representations were learned by the skip-gram algorithm using an adaptive context window. Then, patient representations were constructed based on feature representations weighted by the feature importance. Finally, the proposed patient representation was applied in the mortality risk prediction for acute myocardial infarction in-patients from a public data set and a private data set, and compared with reference methods.

In this study, we aimed to represent various structured features extracted from EMR data as fixed-length embedding vectors, which were then used to improve the performance of predictive models for the death risk of AMI patients. Specifically, we introduced the association strengths into the skip-gram algorithm to learn more informative representations of features. We also introduced the Shapley additive explanations (SHAP) [32] technique to facilitate representation at the patient level and enhance the interpretability of the predictive model. An overview of our proposed representation learning framework and its application is shown in Figure 1.

## Methods

### Skip-Gram–Based Patient Representation

The representation was learned hierarchically at the following 3 levels: the concept, feature, and patient levels. At the concept level, we employed the improved skip-gram algorithm [23] to represent a concept as an embedding vector. In the natural language processing domain, the basic idea of skip-gram was to maximize the occurrence probabilities of the target words and the context words in the predefined context window, making the words that appear in the same context window closer in the embedding space. Unlike words with natural orders in a sentence, medical concepts appeared out of order in a patient record for a certain hospital stay. This made it difficult to determine the context window that contained relevant concepts for the target concept, especially when the number of concepts in a record was far larger than the size of the context window. Thus, for a concept within a record, we identified relevant concepts using its association strength with a candidate concept in the same record. The association strength was defined as the confidence (equation 1) of an association rule with one candidate concept as the unique antecedent (or consequent).

**Figure 2:**
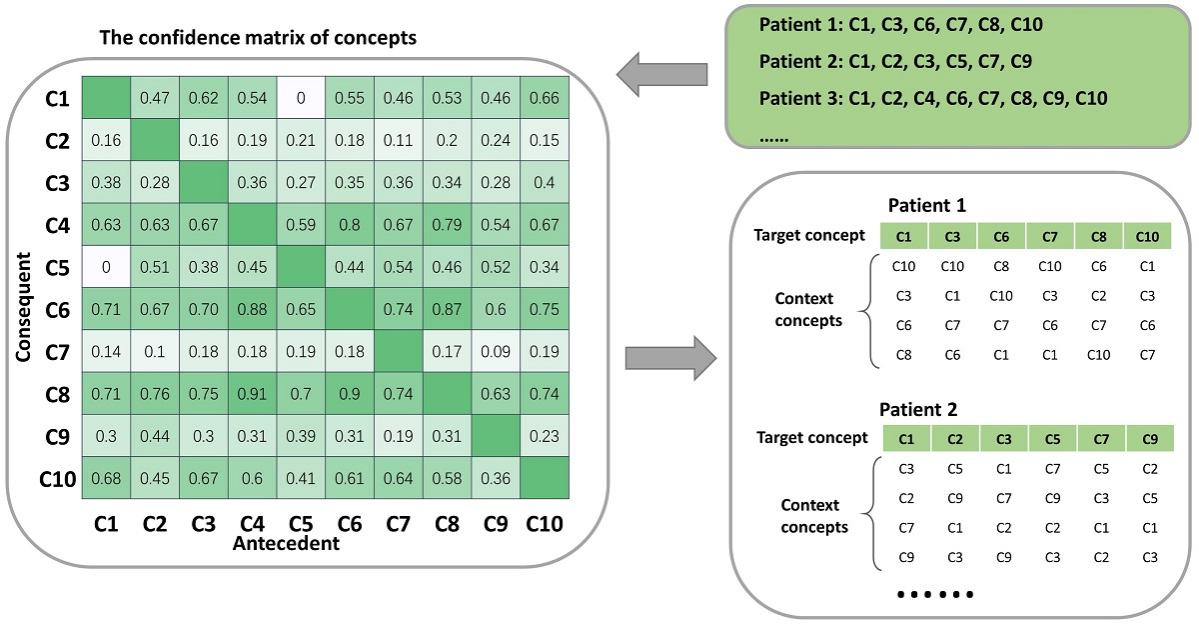
An illustration of context concept selection for the skip-gram algorithm using association strengths. All records are composed of 10 concepts (C1, C2, ……, and C10). In the confidence matrix, element Cij was the confidence of the association rule with Cj as antecedent and Ci as consequent. For patient 1 with 6 concepts (C1, C3, C6, C7, C8, and C10), the included concepts in C1’s 4-concept context window were selected from the remining 5 candidate concepts, whose confidences were 0.66 (antecedent, C10), 0.62 (C3), 0.55 (C6), 0.53 (C8), and 0.46 (C7). Therefore, C10, C3, C6, and C8 were selected to construct the context window for C1.

Confidence (C1, C2) = |C1∩C2| / |C1| **(1)**

where C1 and C2 are the antecedent and consequent concepts, respectively, of an association rule C1→C2, and |C1| and |C1∩C2| are the numbers of patient records containing C1 and both C1 and C2, respectively. The greater the confidence, the stronger the association between the 2 concepts. Antecedent (or consequent) concepts in association rules with the top N highest confidences were included in the context window of the target concept. We called these selection schemes of context concepts *antecedent-based* (or *consequent-based*) embeddings. Figure 2 provides an example of the consequent-based selection scheme of context concepts.

Moreover, to reduce the high dimensionality and sparsity of a large number of concepts, while preserving the clinical information as much as possible, we aggregated the concepts of disease diagnoses and procedures into several clinically meaningful feature groups according to International Classification of Diseases, 10th revision (ICD-10) codes and International Classification of Diseases, 9th revision (ICD-9) codes, and with the help of clinical experts. For example, disease diagnoses of type 1 diabetes mellitus and type 2 diabetes mellitus were grouped into the feature group of diabetes mellitus. The average of all embedding vectors of concepts from the same feature group in a patient record was treated as the representation at the feature level for the patient.

The representation at the patient level was the weighted sum of feature-level representations. The feature weights were obtained under the guidance of the predictive task, indicating the importance of each feature involved in the patient representation. In this study, we used SHAP values as the feature weights. The SHAP framework is a machine learning interpretation technique based on the idea of game theory. It approximated a trained prediction model with a different but simple model that could easily calculate the contribution in the form of a SHAP value for each feature in the prediction model and performed additive feature attribution to explain the combination of features [32]. A positive or negative SHAP value reflected a positive or negative influence on the prediction. A feature’s importance was then computed as the average of its absolute SHAP values from all samples.

### Experiments and Evaluations

#### Data Sets and Data Preprocessing

In this study, we used a public data set, the freely accessible critical care database Medical Information Mart for Intensive Care III (MIMIC-III data set [33]), and a private data set for the experiments.

The MIMIC-III data set was collected between June 2001 and October 2012, and involved 46,520 patients admitted to intensive care units at the Beth Israel Deaconess Medical Center in Boston, Massachusetts. It includes patient health information, such as demographics, vital signs, laboratory test results, medications, procedures, diagnosis codes, and clinical notes. The informative MIMIC-III data set was widely used in some medical machine learning modeling and algorithm evaluations, providing strong data support for researchers to establish models and evaluate algorithms [14,18].

The private data set was derived from the EMR system of a tertiary hospital, Xuanwu Hospital, Capital Medical University, Beijing, China, between January 2014 and December 2016. Patient features included hospital admission and discharge information, demographic data, disease diagnoses, laboratory tests, examinations and procedures, medications, and radiology reports of chest X-ray or color sonography examination.

We extracted the records of all 3010 and 1671 AMI patients from the public and private data sets, respectively. The diagnosis of AMI was confirmed with the ICD-9 codes 410.01 to 410.91 or ICD-10 codes I21 and I22. There were 254 (8.1%) and 103 (6.2%) patients who died in the hospital from the public and private data sets, respectively.

We maintained patients’ first hospitalization data to evaluate the proposed method. Demographic data (age and gender) and the following AMI-related features were maintained in both data sets: AMI-relevant items of laboratory tests that at least 95% of patients carried out, AMI-relevant radiological features extracted from radiology reports [34], 7 commonly prescribed medications, and all recorded disease diagnoses and procedures. For laboratory tests performed more than once, only the results obtained in the first test (usually at admission) were retained, which could reflect a patient’s health status and the severity of illness.

Since initially proposed in the field of natural language processing, the skip-gram algorithm was used to train embeddings for discrete words or symbols. Therefore, to use the skip-gram algorithm for the embedding representation of the structured data, all patient features should be categorical, where each discrete value is treated as a concept. For example, male and female were 2 concepts for gender. Different from raw categorical features, such as gender, disease diagnoses, procedures, and medications, that might remain unchanged, the continuous variables age and laboratory test results had to be discretized into two or more concepts. Age was discretized into 2 concepts (>60 years and ≤60 years). Each laboratory test result was also discretized into 2 concepts (normal and abnormal with reference to clinical standards). In total, 3326 and 1073 medical concepts were identified and further aggregated to 104 and 108 feature groups in the public and private data sets, respectively (Table 1). All feature groups of the private and public data sets are listed in Multimedia Appendix 1 and Multimedia Appendix 2, respectively.

**Table 1:** Concepts and feature groups of both the public and private data sets.

Feature category	Public data set			Private data set			Concept examples
	Feature groups (n=104), n	Concepts (n=3326), n	Feature groups (n=108), n		Concepts (n=1073), n		
Age	1	2	1		2	>60 years and ≤60 years	
Gender	1	2	1		2	Male and female	
Laboratory tests	19	38	40		80	Abnormal serum triglyceride and normal serum creatinine	
Radiological features	34	34	36		36	Cardiac image enlargement and sharp costophrenic angle	
Disease diagnoses	24	2600	15		739	Hypertension and brainstem infarction	
Procedures	18	643	8		207	Coronary stenting and pericardiocentesis	
Medications	7	7	7		7	Angiotensin-converting enzyme inhibitor and heparin	

#### Representation Evaluation

To evaluate the effectiveness and advantages of the proposed representation, we used 2 additional kinds of simple reference representation methods, namely, the 3-layer autoencoder with learning and the feature selection method without learning. Table 2 describes the details of the proposed and reference representation methods.

The proposed representation method was first evaluated at the concept level. Cluster analyses were used to cluster laboratory test concepts into 2 clusters for the quantitative evaluation. The adjusted Rand index (ARI) [35] (ranging from −1 to 1) was used to evaluate the cluster solutions. Greater ARI values indicated higher ability of discriminating from categories with different real labels (normal and abnormal). We also applied the t-distributed stochastic neighbor algorithm to project the embedding vectors of laboratory test concepts into a 2-dimensional space to visually observe the distribution of embeddings.

The proposed representation method was then evaluated at the patient level with a downstream prediction task using the logistic regression model. The predicted outcome was the in-hospital death of AMI patients during hospital stay. The input for prediction was the patient representation derived from the entire feature set listed in Table 1. We also extracted a treatment-free feature subset that excluded medications and procedures from the entire feature set, trying to clarify that the performance of the proposed patient representation was related to the features that were involved in the representation and that the treatment-related features played a crucial role in predicting patient outcome even if they had been represented as embedding vectors.

**Table 2:** Descriptions of the proposed and reference representation methods.

Representation method	Descriptions	Representation examples
Mixture	The mixture of discretization codes for original discrete features and original values for continuous features. The missing values in the laboratory tests were interpolated using the mean of the corresponding laboratory tests.	(0,1,1,0,0,0,1,12,8.5,3,8) for a patient with 11 features
Discretization	The 0-1 vector where the digit 1 represented the patient having the specific disease, procedure, radiological feature, and medication, and 0 otherwise. Age of 1 meant >60 years and 0 meant ≤60 years, gender of 1 meant male and 0 meant female, and a laboratory test item of 1 meant abnormal and 0 meant normal. Missing values for laboratory tests were interpolated by the corresponding mode.	(0,1,1,0,0,0,1,1,0,1,1) for a patient with 11 discretization features
DIS_FS^a^	The selected features with discretization representations were statistically different between patients with and without the label “death.”	(0,0,1,0,0,1,0,1) for a patient with 8 selected features
DIS_AE^b^	The hidden-layer vector of a 3-layer autoencoder with discretization vectors as inputs and outputs. The dimension of the hidden layer was set to 64.	(0.7,1.9,0.5,−1,−3.1,2.4) for a patient with a 6-dimensional vector
RAN_EM_AVE^c^	The average of feature embedding vectors learned from the skip-gram algorithm using the random selection method to determine the context window.	(1.6,−0.5,1.1,0.1,−1.3,0.6) for a patient with a 6-dimensional embedding vector
RAN_EM_WGT^d^	The weighted sum of the feature embedding vectors learned from the skip-gram algorithm using the random selection method to determine the context window.	(1.2,−0.9,1.3,0.4,−1.9,1.0) for a patient with a 6-dimensional embedding vector
ANT_EM_AVE^e^	The average of the feature embedding vectors learned from the skip-gram algorithm using the confidence with the target concept as the antecedent.	(0.9,−0.6,1.2,1.4,−1.9,0.6) for a patient with a 6-dimensional embedding vector
ANT_EM_WGT^f^	The weighted sum of the feature embedding vectors learned from the skip-gram algorithm using the confidence with the target concept as the antecedent.	(1.2,−1.5,1.1,0.1,−0.6,0.6) for a patient with a 6-dimensional embedding vector
CON_EM_AVE^g^	The average of the feature embedding vectors learned from the skip-gram algorithm using the confidence with the target concept as the consequent.	(1.6,−0.8,2.1,1.6,−1.4,1.5) for a patient with a 6-dimensional embedding vector
CON_EM_WGT^h^	The weighted sum of the feature embedding vectors learned from the skip-gram algorithm using the confidence with the target concept as the consequent.	(1.1,−0.4,−0.7,1.6,−0.3,0.9) for a patient with a 6-dimensional embedding vector

^a^DIS_FS: discretization representations with feature selection.

^b^DIS_AE: hidden vector of an autoencoder-based representation.

^c^RAN_EM_AVE: average of the random selection–based embedding representation.

^d^RAN_EM_WGT: weighted sum of the random selection–based embedding representation.

^e^ANT_EM_AVE: average of the antecedent-based embedding representation.

^f^ANT_EM_WGT: weighted sum of the antecedent-based embedding representation.

^g^CON_EM_AVE: average of the consequent-based embedding representation.

^h^CON_EM_WGT: weighted sum of the consequent-based embedding representation.

We randomly split samples into training and test data sets by the ratio of 7:3. The training samples were first represented in the discretization vectors and used to build a predictive model for calculating all features’ SHAP values for the further patient embedding representations of all study samples. After being represented as embedding vectors, the training and test samples were used to build and validate a logistic regression-based predictive model, respectively. The area under the receiver operating characteristic curve (AUROC), area under the precision-recall curve (AUPRC), and F1-score were the main evaluation metrics. Other relevant performance metrics from the confusion matrix included precision, recall, and accuracy. To eliminate the performance bias introduced by the initialization of a skip-gram model and the training/test data set split, we performed the comparative experiment 100 times. In each experiment round, the above processes were repeated. The mean with its 95% CI of each performance evaluation metric was reported.

In the skip-gram algorithm, the size of the context window and the dimension of the embedding vector were determined by trial and error. We conducted a group of predictive experiments on the public data set, using possible combinations of window sizes of 5, 10, 15, and 20, and vector dimensions of 50, 100, 200, and 300. Experimental results (listed in Multimedia Appendix 3) showed that the skip-gram algorithm with the combination of a window size of 10 and a vector dimension of 300 had the highest representation performance. Therefore, the size of the context window and the dimension of the embedding vector were set to 10 and 300, respectively. We applied the negative sampling mechanism (20 negative samples in this study) to accelerate the concept embedding training process. Other parameters were as follows: learning rate, 0.001; number of iterations, 50; batch size, 64. The gradient calculation method was Adam. We implemented representation learning, SHAP value computation, and prediction modeling in Python 3.7 and TensorFlow 2.0. In the step of patient representation, we used the L2 regularization penalty with “liblinear” solver for the logistic regression model, and the inverse of regularization strength was set to 0.1.

### Ethics Approval

The study was approved by the Human Research Ethics Committees of Xuanwu Hospital, Capital Medical University (approval number: Clinical Scientific Research 2020-070).

## Results

### Concept Representation Evaluation

**Figure 3:**
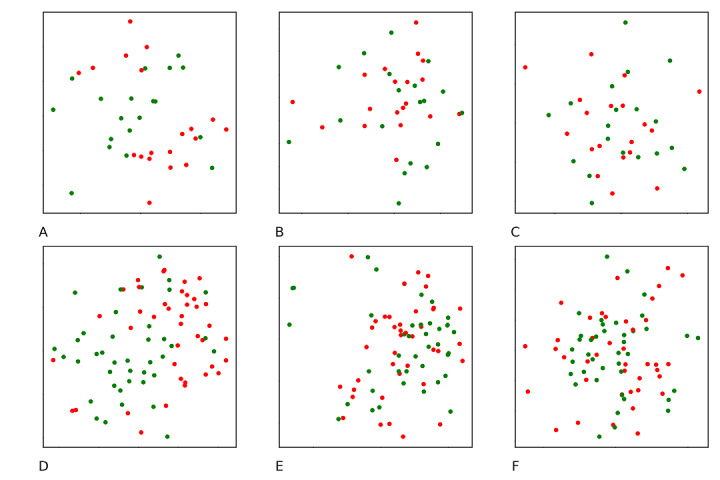
Visualization of the embedding laboratory tests using different selection schemes for contextual concepts in the skip-gram algorithm (the t-distributed stochastic neighbor embedding algorithm was used). Dots in red and green represent abnormal and normal laboratory test results, respectively. A to C for the public data set: the contextual concepts of a target concept consist of its consequent concepts (A) or antecedent concepts (B) in association rules, or randomly selected concepts (C). D to F are the counterparts of A to C on the private data set.

Embedding vectors for laboratory test concepts were visualized in a plane space (Figure 3). Concepts of normal and abnormal laboratory tests (Figure 3) were farther away when they were represented by the consequent-based embeddings (Figures 3A and 3D) than by the antecedent-based embeddings (Figures 3B and 3E) and the random selection–based embeddings (Figures 3C and 3F). In cluster analyses for laboratory tests, the consequent-based embeddings achieved higher ARIs (0.317 and 0.520 on the public and private data sets, respectively) than the antecedent-based embeddings (0.112 and 0.149, respectively) and the random selection–based embeddings (0.043 and 0.028, respectively). The best cluster performance of the consequent-based embeddings among the 3 embeddings indicated that the consequent-based embeddings might contain more feature association information.

### Predictive Performance

Table 3 and Multimedia Appendix 4 list the predictive performances using various representation methods on the private and public data sets, respectively. The proposed representation method, the weighted sum of the consequent-based embedding representation (CON_EM_WGT), showed the highest predictive performances, with maximum AUROCs of 0.878, 0.973, and 0.926 using all features of the public data set and the entire and treatment-free feature sets of the private data set, respectively. When the performance was measured by AUPRC and F1-score, the proposed representation method outperformed all the other methods regardless of the data sets and feature sets.

Compared with the reference representations, most of the embedding-based representations on both data sets showed a performance improvement. The average AUROC, AUPRC, and F1-score of the 6 representation methods with embeddings were greater than those of the 4 reference methods without embeddings (0.855 vs 0.831, 0.203 vs 0.185, and 0.354 vs 0.328, respectively) on the public data set with the entire feature set. Further, among the 6 representations based on the skip-gram algorithm, representations with algorithm improvement based on the association strength achieved superior performance than those without.

When assembling feature representations into a patient representation, the assembling method and the involved features did matter. Representations based on the idea of weighted sum outperformed those based on the idea of average, on either the public data set with the entire feature set (AUROC, 0.863 to 0.878 vs 0.834 to 0.850) or the private data set with the entire feature set (0.967 to 0.973 vs 0.948 to 0.957). On the other hand, consistently superior predictive performance was achieved on both data sets with the entire feature set compared with the treatment-free feature set. Multimedia Appendix 5 shows the average predictive performance of patient representation methods on the public and private data sets with and without treatment feature sets.

**Table 3:** Predictive performance of patient representation methods on the private data set.

Feature set and representation methods				AUROC^a^, mean (95% CI)		AUPRC^b^, mean (95% CI)		F1-score, mean (95% CI)
**Entire feature set**								
	**Embedding-based representation methods**							
		CON_EM_WGT^c^	0.973 (0.951-0.995)		0.505 (0.278-0.732)		0.674 (0.468-0.880)	
		CON_EM_AVE^d^	0.957 (0.933-0.981)		0.312 (0.159-0.465)		0.479 (0.301-0.657)	
		ANT_EM_WGT^e^	0.972 (0.948-0.996)		0.489 (0.258-0.720)		0.658 (0.442-0.874)	
		ANT_EM_AVE^f^	0.953 (0.929-0.977)		0.310 (0.185-0.435)		0.478 (0.329-0.627)	
		RAN_EM_WGT^g^	0.967 (0.942-0.992)		0.486 (0.263-0.709)		0.660 (0.460-0.860)	
		RAN_EM_AVE^h^	0.948 (0.923-0.973)		0.287 (0.167-0.407)		0.451 (0.306-0.596)	
	**Reference representation methods**							
		DIS_AE^i^	0.884 (0.845-0.923)		0.207 (0.144-0.270)		0.361 (0.279-0.443)	
		DIS_FS^j^	0.938 (0.907-0.969)		0.283 (0.167-0.399)		0.452 (0.309-0.595)	
		Discretization	0.939 (0.908-0.970)		0.283 (0.165-0.401)		0.454 (0.307-0.601)	
		Mixture	0.904 (0.849-0.959)		0.251 (0.135-0.367)		0.417 (0.264-0.570)	
**Treatment-free feature set**								
	**Embedding-based representation methods**							
		CON_EM_WGT	0.926 (0.883-0.969)		0.282 (0.139-0.425)		0.456 (0.282-0.630)	
		CON_EM_AVE	0.915 (0.876-0.954)		0.248 (0.156-0.340)		0.413 (0.297-0.529)	
		ANT_EM_WGT	0.919 (0.874-0.964)		0.278 (0.133-0.423)		0.455 (0.275-0.635)	
		ANT_EM_AVE	0.912 (0.869-0.955)		0.256 (0.162-0.350)		0.423 (0.307-0.539)	
		RAN_EM_WGT	0.915 (0.868-0.962)		0.248 (0.119-0.377)		0.416 (0.238-0.594)	
		RAN_EM_AVE	0.897 (0.850-0.944)		0.225 (0.133-0.317)		0.385 (0.265-0.505)	
	**Reference representation methods**							
		DIS_AE	0.884 (0.845-0.923)		0.207 (0.144-0.270)		0.361 (0.279-0.443)	
		DIS_FS	0.903 (0.862-0.944)		0.214 (0.124-0.304)		0.367 (0.236-0.498)	
		Discretization	0.905 (0.862-0.948)		0.224 (0.122-0.326)		0.381 (0.238-0.524)	
		Mixture	0.867 (0.806-0.928)		0.202 (0.116-0.288)		0.356 (0.227-0.485)	

^a^AUROC: area under the receiver operating characteristic curve.

^b^AUPRC: area under the precision-recall curve.

^c^CON_EM_WGT: weighted sum of the consequent-based embedding representation.

^d^CON_EM_AVE: average of the consequent-based embedding representation.

^e^ANT_EM_WGT: weighted sum of the antecedent-based embedding representation.

^f^ANT_EM_AVE: average of the antecedent-based embedding representation.

^g^RAN_EM_WGT: weighted sum of the random selection–based embedding representation.

^h^RAN_EM_AVE: average of the random selection–based embedding representation.

^i^DIS_AE: discretization representations with features selection.

^j^DIS_FS: hidden vector of an autoencoder-based representation.

### Predictive Model Interpretation

**Figure 4:**
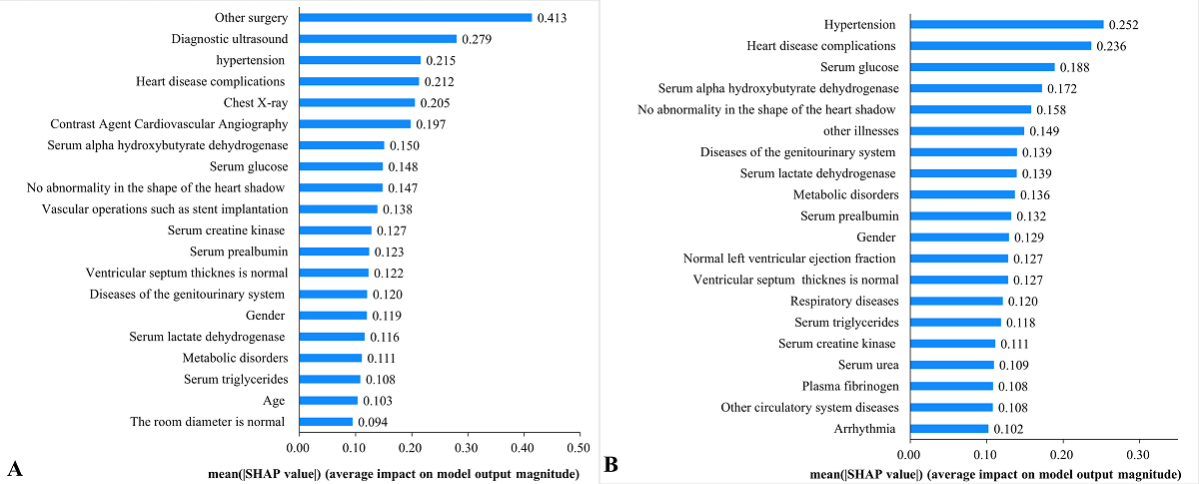
The mean absolute Shapley additive explanations (SHAP) values of the top 20 features of the private data set within the entire feature set (A) and the treatment-free feature set (B).

Figure 4 illustrates the global feature attributions for the top 20 most important features from the private data set when predicting in-hospital death risk. The treatment-related features played an important role in the mortality prediction. These features included other surgery (mean absolute SHAP value: 0.413), diagnostic ultrasound (0.279), contrast agent cardiovascular angiography (0.197), etc (Figure 4A). Moreover, comorbidity diseases like hypertension (mean absolute SHAP value: 0.252) and heart disease complications (0.236), and laboratory tests like serum glucose (0.188) and serum lactate dehydrogenase (0.139) had strong associations with in-hospital death (Figure 4B). SHAP values of features in the public data set are shown in Multimedia Appendix 6.

**Figure 5:**
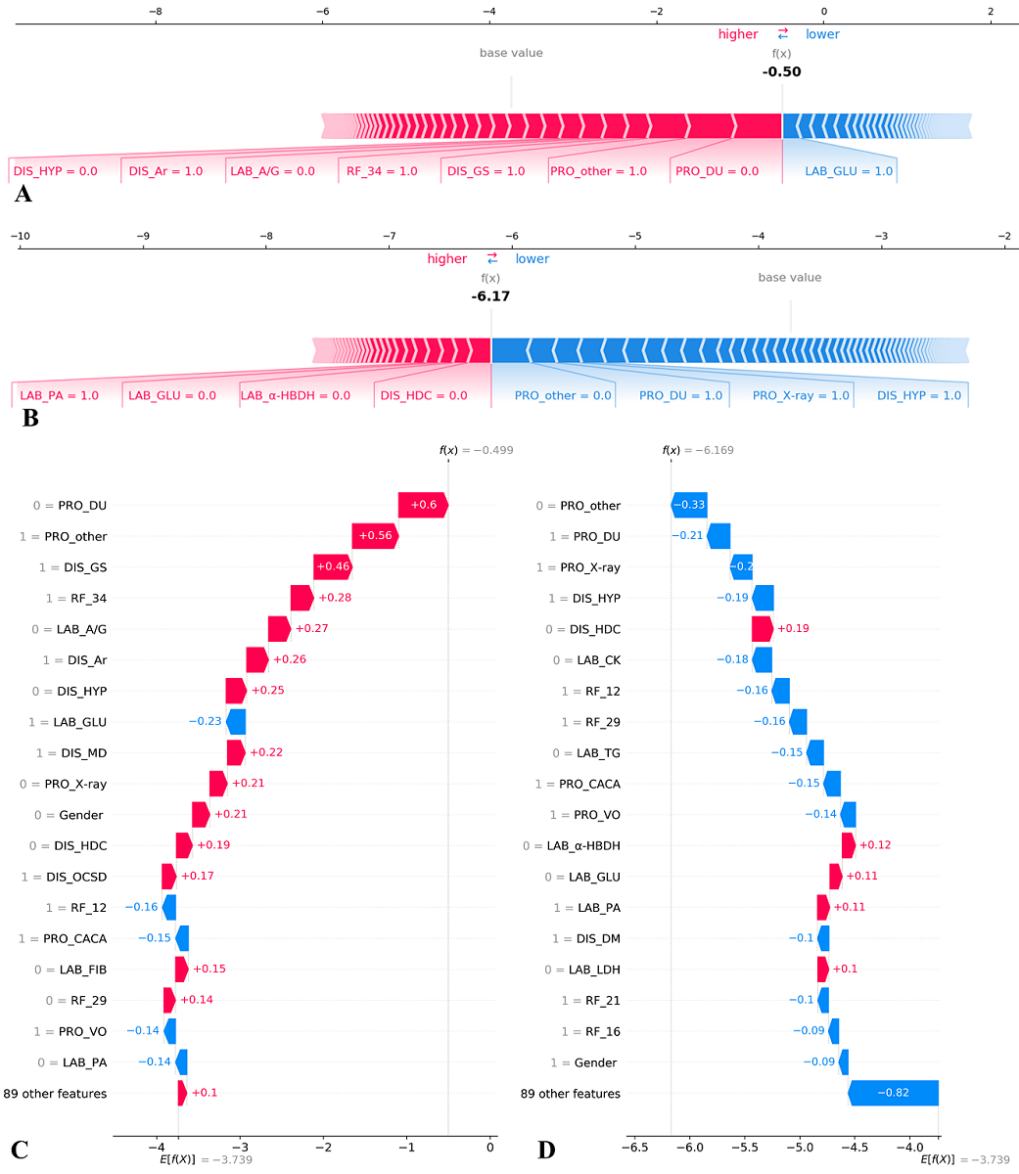
Shapley additive explanations (SHAP) values for a patient who died during hospital stay (A and C) and another patient who did not die (B and D). Both patients were selected from the private data set with the entire feature set. A and B, all features with their SHAP values. C and D, 20 features with the greatest absolute SHAP values. Features in blue tend to reduce the possibility of a patient being classified as positive (death in this study), while features in red do the contrary. The meaning of each abbreviated feature name can be found in Multimedia Appendix 1.

In addition to the feature’s global importance in the specific predictive task, SHAP values were helpful in distinguishing the feature’s local importance, that is, the importance for an individual sample. Figure 5 illustrates how the mortality risk was predicted with SHAP values for a patient who died during hospital stay and another patient who did not die. The positive SHAP values of most features of the patient who died during hospital stay increased the total SHAP value from an average value of −3.739 to a final value of −0.499 (Figures 5A and 5C), meaning that the patient had a higher risk of in-hospital death than the average. In this incremental process, gender as female, for example, contributed a SHAP value of +0.21 (Figures 5C). On the contrary, the negative SHAP values of most features of the patient who was discharged alive decreased the total SHAP value from −3.739 to −6.169 (Figures 5B and 5D), indicating a lower death risk. In this decremental process, male gender contributed a SHAP value of −0.09 (Figures 5D). We have shown 2 examples of patients from the public data set in Multimedia Appendix 7.

## Discussion

### Principal Findings

With the widespread adoption of EMR data in building machine learning–based predictive models, one of the most fundamental research challenges was learning proper patient representations that might capture hidden semantic associations among medical concepts [18]. In this study, we proposed an improved skip-gram–based patient representation method where the association strength among medical concepts and the task-specific feature importance were integrated. Compared with other representation methods, the proposed patient representation improved the performance of the mortality risk prediction for AMI patients.

In previous studies, deep learning models [9,10,12,25,36] were used in training embedding representations of medical concepts for the subsequent patient representation. When using the skip-gram algorithm, the order of medical concepts that was independent of feature relevance hindered the algorithm from learning high-quality representation. Prior work recommended the shuffling mechanism for medical concepts in a patient record to reduce the impact of the out-of-order characteristics on the algorithm [12,24-26]. In this study, we introduced the association strength between 2 concepts that was defined as the confidence of an association rule involving just the 2 concepts. Experiments from various aspects indicated that this ingenious improvement was effective in revealing potential associations among medical concepts and further enhancing the performance of downstream prediction tasks.

In addition to the representation algorithm, features used to represent a patient were also critical. Many previous studies focused on some features in the original form of medical codes, such as disease diagnoses, procedures, and medications [1,11,14,37]. For laboratory tests that contained much diagnosis and prognosis-relevant information about patients, we included the normal status of the laboratory tests into the feature sets, rather than simply using the number of laboratory tests and test co-occurrences [12,38]. We further extracted radiological features from free-text radiological reports. Admittedly, richer features may lead to a feature representation with more information, even if the dimension of patient representation remains unchanged. In this study, predictive models using more features to represent a patient did reflect more information about the patient and showed higher performance than those using fewer features. Our finding is similar to the results of other studies [39,40].

Prior studies employed neural networks to train predictive models for clinical outcomes using EMR data [2,16,22]. They focused on end-to-end prediction models built on large data sets, where the last hidden layer of the neural network was regarded as the patient representation. Although the deep end-to-end neural network–based patient representation improved the predictive accuracy, the lack of interpretability could not be ignored. Some studies [26,41] constructed patient representations using the average of concept representations learned by word embedding methods, which did not make full use of the importance of different clinical features for patients. As an advanced interpretability method, the SHAP value [32] was successfully used to analyze and explain the predictive models in some previous studies [40,42-44]. We introduced SHAP values as feature importance into the patient representation, and further explained the predictive model with SHAP values. SHAP values can be used to not only rank the overall importance and identify the important factors for the prediction task, but also explore the key factors for predicting the mortality risk for a specific patient. In our predictive task for AMI patients, the most important features identified by SHAP values were really closely related to AMI [45-47], such as serum glucose and serum creatine kinase, which are 2 critical laboratory tests for AMI diagnosis and prognosis in clinical practice.

In our predictive task, the model that took all available patient characteristics represented by the proposed patient representation method as inputs showed a higher performance than other models on the same task in previous studies (AUROC, 0.973 vs 0.905 to 0.935 [19,29-31,48]). This may be because the embedding representation contained a large number of diverse features extracted from a general EMR system, while many researchers selected AMI-related features with the assistance of clinical experts. For example, basic demographic data and few laboratory tests, as well as several specific features of AMI like Killip classification and left ventricular ejection fraction [19,30] were directly added into the machine learning model to predict mortality risk. Further, compared with other simple feature extraction methods like Principal Component Analysis [29] and the 3-layer autoencoder model, the proposed method took the association strength and feature importance into consideration, achieving higher predictive performance.

### Limitations

This study had some limitations. First, only patients’ laboratory tests for the first time during hospital stay were included in this study, while many patients took two or more laboratory tests. Since temporal data, especially multiple laboratory tests, may reflect the dynamic health status and the treatment effect of a patient over time, the lack of temporal characteristics of laboratory tests in the patient representation may lead to performance loss in downstream tasks. A future study will focus on integrating this uneven and irregular temporal data into the current patient representation. Second, the skip-gram algorithm was used in training concept embeddings. The algorithm is popular in the natural language processing domain, possibly having a limited ability to represent structured and disordered EMR data. A transformer-based pretrain model, Med-Bert, has been trained to represent disease diagnoses originally expressed in ICD-10 and ICD-9 codes, showing higher performance with AUROCs of 85.39% and 82.23% in heart failure and pancreatic cancer prediction tasks, respectively [49]. Therefore, more complicated deep learning methods will be adopted for a more informative patient representation in the future. Lastly, we carried out only internal validation of the predictive model built on the proposed patient representation. External validation of high quality will be more convincing and will help in continuous algorithm improvement. Moreover, the chosen reference methods for the performance comparison were simple feature selection methods and a 3-layer autoencoder. Comparison with state-of-the-art methods is needed to evaluate the performance and potential use of our proposed method.

### Conclusions

In this study, we improved the embedding-based patient representation with the association strength of medical concepts and importance of patient features. After further training and fine-tuning, the model based on the proposed patient representation will hopefully be used to assist in prognostic prediction for AMI inpatients. This study puts forward a meaningful direction for the development of more effective and efficient clinical prediction models using EMR data. It is desirable for patient representation learning to serve as an essential part of building a predictive model for clinical outcomes.

## Appendix

Multimedia Appendix 1 Patient features of samples in the private data set.

Multimedia Appendix 2 Patient features of samples in the public MIMIC-III data set.

Multimedia Appendix 3 Predictive performance of skip-gram–based embedding representations with different combinations of the size of the context window and the dimension of the embedding vector.

Multimedia Appendix 4 Predictive performance of patient representation methods on the public data set.

Multimedia Appendix 5 The average predictive performance of patient representation methods on the public and private data sets with and without treatment feature sets.

Multimedia Appendix 6 The mean absolute Shapley additive explanations (SHAP) values of the top 20 features of the public data set within the entire feature set (A) and the treatment-free feature set (B).

Multimedia Appendix 7 Shapley additive explanations (SHAP) values for a patient who died during hospital stay (A and C) and another patient who did not die (B and D) from the public data set with the entire feature set.

## Abbreviations

AMI: acute myocardial infarction
ARI: adjusted Rand index
AUPRC: area under the precision-recall curve
AUROC: area under the receiver operating characteristic curve
EMR: electronic medical record
ICD: International Classification of Diseases
SHAP: Shapley additive explanations

## References

[1] Xiao X, Wei G, Zhou L, Pan Y, Jing H, Zhao E, Yuan Y (2021). Treatment initiation prediction by EHR mapped PPD tensor based convolutional neural networks boosting algorithm. J Biomed Inform.

[2] Li L, Jiang Y, Huang B (2021). Long-term prediction for temporal propagation of seasonal influenza using Transformer-based model. J Biomed Inform.

[3] Ma H, Sheng W, Li J, Hou L, Yang J, Cai J, Xu W, Zhang S (2021). A novel hierarchical machine learning model for hospital-acquired venous thromboembolism risk assessment among multiple-departments. J Biomed Inform.

[4] Tang Z, Yu Y, Ng K, Sow D, Hu J, Mei J (2021). Disease network delineates the disease progression profile of cardiovascular diseases. J Biomed Inform.

[5] Chen P, Dong W, Lu X, Kaymak U, He K, Huang Z (2019). Deep representation learning for individualized treatment effect estimation using electronic health records. J Biomed Inform.

[6] Dligach D, Afshar M, Miller T (2019). Toward a clinical text encoder: pretraining for clinical natural language processing with applications to substance misuse. J Am Med Inform Assoc.

[7] Kamal SA, Yin C, Qian B, Zhang P (2020). An interpretable risk prediction model for healthcare with pattern attention. BMC Med Inform Decis Mak.

[8] Zhang X, Chou J, Liang J, Xiao C, Zhao Y, Sarva H, Henchcliffe C, Wang F (2019). Data-driven subtyping of Parkinson's disease using longitudinal clinical records: A cohort study. Sci Rep.

[9] Luo X, Gandhi P, Zhang Z, Shao W, Han Z, Chandrasekaran V, Turzhitsky V, Bali V, Roberts AR, Metzger M, Baker J, La Rosa C, Weaver J, Dexter P, Huang K (2021). Applying interpretable deep learning models to identify chronic cough patients using EHR data. Comput Methods Programs Biomed.

[10] Li Z, Roberts K, Jiang X, Long Q (2019). Distributed learning from multiple EHR databases: Contextual embedding models for medical events. J Biomed Inform.

[11] Barbieri S, Kemp J, Perez-Concha O, Kotwal S, Gallagher M, Ritchie A, Jorm L (2020). Benchmarking deep learning architectures for predicting readmission to the ICU and describing patients-at-risk. Sci Rep.

[12] Steinberg E, Jung K, Fries JA, Corbin CK, Pfohl SR, Shah NH (2021). Language models are an effective representation learning technique for electronic health record data. J Biomed Inform.

[13] Carrasco-Ribelles LA, Pardo-Mas JR, Tortajada S, Sáez C, Valdivieso B, García-Gómez JM (2021). Predicting morbidity by local similarities in multi-scale patient trajectories. J Biomed Inform.

[14] Yang S, Zheng X, Ji C, Chen X (2021). Multi-layer representation learning and its application to electronic health records. Neural Process Lett.

[15] Wang Z, Wang B, Zhou Y, Li D, Yin Y (2020). Weight-based multiple empirical kernel learning with neighbor discriminant constraint for heart failure mortality prediction. J Biomed Inform.

[16] Rongali S, Rose AJ, McManus DD, Bajracharya AS, Kapoor A, Granillo E, Yu H (2020). Learning latent space representations to predict patient outcomes: Model development and validation. J Med Internet Res.

[17] Tahayori B, Chini-Foroush N, Akhlaghi H (2021). Advanced natural language processing technique to predict patient disposition based on emergency triage notes. Emerg Med Australas.

[18] Si Y, Du J, Li Z, Jiang X, Miller T, Wang F, Jim Zheng W, Roberts K (2021). Deep representation learning of patient data from Electronic Health Records (EHR): A systematic review. J Biomed Inform.

[19] Kwon J, Jeon K, Kim HM, Kim MJ, Lim S, Kim K, Song PS, Park J, Choi RK, Oh B (2019). Deep-learning-based risk stratification for mortality of patients with acute myocardial infarction. PLoS One.

[20] Ruan T, Lei L, Zhou Y, Zhai J, Zhang L, He P, Gao J (2019). Representation learning for clinical time series prediction tasks in electronic health records. BMC Med Inform Decis Mak.

[21] Zhao J, Papapetrou P, Asker L, Boström H (2017). Learning from heterogeneous temporal data in electronic health records. J Biomed Inform.

[22] Morid MA, Sheng ORL, Kawamoto K, Abdelrahman S (2020). Learning hidden patterns from patient multivariate time series data using convolutional neural networks: A case study of healthcare cost prediction. J Biomed Inform.

[23] Mikolov T, Sutskever I, Chen K, Corrado G, Dean J (2013). Distributed representations of words and phrases and their compositionality. NIPS'13: Proceedings of the 26th International Conference on Neural Information Processing Systems - Volume 2.

[24] Glicksberg BS, Miotto R, Johnson KW, Shameer K, Li L, Chen R, Dudley JT (2018). Automated disease cohort selection using word embeddings from Electronic Health Records. Pac Symp Biocomput.

[25] Cui L, Xie X, Shen Z (2018). Prediction task guided representation learning of medical codes in EHR. J Biomed Inform.

[26] Huang Y, Wang N, Zhang Z, Liu H, Fei X, Wei L, Chen H (2021). Patient representation from structured electronic medical records based on embedding technique: Development and validation study. JMIR Med Inform.

[27] Chen H, Shi L, Xue M, Wang N, Dong X, Cai Y, Chen J, Zhu W, Xu H, Meng Q (2018). Geographic variations in in‐hospital mortality and use of percutaneous coronary intervention following acute myocardial infarction in China: A nationwide cross‐sectional analysis. J Am Heart Assoc.

[28] GBD 2013 Mortality Causes of Death Collaborators (2015). Global, regional, and national age–sex specific all-cause and cause-specific mortality for 240 causes of death, 1990–2013: a systematic analysis for the Global Burden of Disease Study 2013. The Lancet.

[29] Lee HC, Park JS, Choe JC, Ahn JH, Lee HW, Oh J, Choi JH, Cha KS, Hong TJ, Jeong MH, Korea Acute Myocardial Infarction Registry (KAMIR) Korea Working Group on Myocardial Infarction (KorMI) Investigators (2020). Prediction of 1-year mortality from acute myocardial infarction using machine learning. Am J Cardiol.

[30] Aziz F, Malek S, Ibrahim KS, Raja Shariff RE, Wan Ahmad WA, Ali RM, Liu KT, Selvaraj G, Kasim S (2021). Short- and long-term mortality prediction after an acute ST-elevation myocardial infarction (STEMI) in Asians: A machine learning approach. PLoS One.

[31] Wang Q, Qian W, Sun Z, Zhu W, Liu Y, Chen X, Ji Y, Sun L (2020). Nomograms based on pre-operative parametric for prediction of short-term mortality in acute myocardial infarction patients treated invasively. Aging (Albany NY).

[32] Lundberg SM, Lee SI (2017). A unified approach to interpreting model predictions. NIPS'17: Proceedings of the 31st International Conference on Neural Information Processing Systems.

[33] Medical Information Mart for Intensive Care.

[34] Wang N, Wang M, Zhou Y, Liu H, Wei L, Fei X, Chen H (2022). Sequential data-based patient similarity framework for patient outcome prediction: Algorithm development. J Med Internet Res.

[35] Xie J, Gao H, Xie W, Liu X, Grant PW (2016). Robust clustering by detecting density peaks and assigning points based on fuzzy weighted K-nearest neighbors. Information Sciences.

[36] Wang L, Wang Q, Bai H, Liu C, Liu W, Zhang Y, Jiang L, Xu H, Wang K, Zhou Y (2020). EHR2Vec: Representation learning of medical concepts from temporal patterns of clinical notes based on self-attention mechanism. Front Genet.

[37] Bai T, Chanda AK, Egleston BL, Vucetic S (2018). EHR phenotyping via jointly embedding medical concepts and words into a unified vector space. BMC Med Inform Decis Mak.

[38] Miotto R, Li L, Kidd BA, Dudley JT (2016). Deep patient: An unsupervised representation to predict the future of patients from the electronic health records. Sci Rep.

[39] Zhang D, Yin C, Zeng J, Yuan X, Zhang P (2020). Combining structured and unstructured data for predictive models: a deep learning approach. BMC Med Inform Decis Mak.

[40] Xu Y, Liu X, Pan L, Mao X, Liang H, Wang G, Chen T (2022). Explainable dynamic multimodal variational autoencoder for the prediction of patients with suspected central precocious puberty. IEEE J. Biomed. Health Inform.

[41] Choi E, Schuetz A, Stewart WF, Sun J (2016). Medical concept representation learning from electronic health records and its application on heart failure prediction. arXiv.

[42] Müller M, Gromicho M, de Carvalho M, Madeira SC (2021). Explainable models of disease progression in ALS: Learning from longitudinal clinical data with recurrent neural networks and deep model explanation. Computer Methods and Programs in Biomedicine Update.

[43] Lundberg SM, Erion G, Chen H, DeGrave A, Prutkin JM, Nair B, Katz R, Himmelfarb J, Bansal N, Lee S (2020). From local explanations to global understanding with explainable AI for trees. Nat Mach Intell.

[44] Lundberg SM, Nair B, Vavilala MS, Horibe M, Eisses MJ, Adams T, Liston DE, Low DKW, Newman S, Kim J, Lee S (2018). Explainable machine-learning predictions for the prevention of hypoxaemia during surgery. Nat Biomed Eng.

[45] Pinto DS, Grandin EW (2017). Risk prediction in AMI shock: Goldilocks and the search for "just right". J Am Coll Cardiol.

[46] Yeh RW, Sidney S, Chandra M, Sorel M, Selby JV, Go AS (2010). Population trends in the incidence and outcomes of acute myocardial infarction. N Engl J Med.

[47] Shroff GR, Frederick PD, Herzog CA (2012). Renal failure and acute myocardial infarction: clinical characteristics in patients with advanced chronic kidney disease, on dialysis, and without chronic kidney disease. A collaborative project of the United States Renal Data System/National Institutes of Health and the National Registry of Myocardial Infarction. Am Heart J.

[48] D'Ascenzo F, De Filippo O, Gallone G, Mittone G, Deriu M, Iannaccone M, Ariza-Solé A, Liebetrau C, Manzano-Fernández S, Quadri G, Kinnaird T, Campo G, Simao Henriques J, Hughes J, Dominguez-Rodriguez A, Aldinucci M, Morbiducci U, Patti G, Raposeiras-Roubin S, Abu-Assi E, De Ferrari G, Piroli F, Saglietto A, Conrotto F, Omedé P, Montefusco A, Pennone M, Bruno F, Bocchino P, Boccuzzi G, Cerrato E, Varbella F, Sperti M, Wilton S, Velicki L, Xanthopoulou I, Cequier A, Iniguez-Romo A, Munoz Pousa I, Cespon Fernandez M, Caneiro Queija B, Cobas-Paz R, Lopez-Cuenca A, Garay A, Blanco P, Rognoni A, Biondi Zoccai G, Biscaglia S, Nunez-Gil I, Fujii T, Durante A, Song X, Kawaji T, Alexopoulos D, Huczek Z, Gonzalez Juanatey J, Nie S, Kawashiri M, Colonnelli I, Cantalupo B, Esposito R, Leonardi S, Grosso Marra W, Chieffo A, Michelucci U, Piga D, Malavolta M, Gili S, Mennuni M, Montalto C, Oltrona Visconti L, Arfat Y (2021). Machine learning-based prediction of adverse events following an acute coronary syndrome (PRAISE): a modelling study of pooled datasets. The Lancet.

[49] Rasmy L, Xiang Y, Xie Z, Tao C, Zhi D (2021). Med-BERT: pretrained contextualized embeddings on large-scale structured electronic health records for disease prediction. NPJ Digit Med.

