# Supplementary material for: Improving the Performance of Outcome Prediction for Inpatients With Acute Myocardial Infarction Based on Embedding Representation Learned From Electronic Medical Records: Development and Validation Study
Source: J Med Internet Res. 2022 Aug 3;24(8):e37486. doi: 10.2196/37486 (PMC9386580; doi:10.2196/37486)
Supplement: Multimedia Appendix 1 [file jmir_v24i8e37486_app1.docx]

**Multimedia Appendix 1.** Patient features of samples in the private data set.

| Feature categories | ID | Feature groups | Abbreviation | Mean±Standard deviation or N(%) |
| --- | --- | --- | --- | --- |
| Demographic features | 1 | Age, year | Age | 64.53±12.72 |
|  | 2 | Gender, male | Gender | 1222(73.13%) |
| Laboratory tests | 3 | Serum α-hydroxybutyrate dehydrogenase, IU/L | LAB_α-HBDH | 365.29±320.01 |
|  | 4 | Serum γ-glutamyl transpeptidase, IU/L | LAB_GGT | 35.87±38.59 |
|  | 5 | Serum Albumin / globulin | LAB_A/G | 1.63±0.34 |
|  | 6 | Serum albumin, g/L | LAB_ALB | 37.84±4.32 |
|  | 7 | Serum alanine aminotransferase , IU/L | LAB_ALT | 35.92±64.45 |
|  | 8 | Serum low density lipoprotein, mmol/L | LAB_LDL | 2.68±0.87 |
|  | 9 | Serum calcium, mmol/L | LAB_Ca | 2.12±0.14 |
|  | 10 | Serum triglycerides, mmol/L | LAB_TG | 1.83±1.46 |
|  | 11 | Serum high density lipoprotein, mmol/L | LAB_HDL | 1.24±0.33 |
|  | 12 | Serum creatinine, umol/L | LAB_Cr | 80.99±56.66 |
|  | 13 | Serum creatine kinase , IU/L | LAB_CK | 744.75±1217.97 |
|  | 14 | Serum potassium, mmol/L | LAB_K | 4.06±0.54 |
|  | 15 | Serum indirect bilirubin , umol/L | LAB_I-Bil | 9.59±4.61 |
|  | 16 | Serum alkaline phosphatase , IU/L | LAB_ALP | 67.24±31.44 |
|  | 17 | Serum phosphorus, mmol/L | LAB_Pi | 1.04±0.24 |
|  | 18 | Serum chlorine, mmol/L | LAB_Cl | 102.46±3.86 |
|  | 19 | Serum sodium, mmol/L | LAB_Na | 139.92±3.51 |
|  | 20 | Serum urea, mmol/L | LAB_Ure | 6.70±3.41 |
|  | 21 | Serum uric acid, umol/L | LAB_URIC | 332.17±102.07 |
|  | 22 | Serum glucose, mmol/L | LAB_GLU | 8.75±4.31 |
|  | 23 | Serum prealbumin, mg/L | LAB_PA | 207.10±59.70 |
|  | 24 | Serum globulin, g/L | LAB_GLB | 23.89±4.05 |
|  | 25 | Serum lactate dehydrogenase, IU/L | LAB_LDH | 398.65±311.17 |
|  | 26 | Serum aspartate aminotransferase, IU/L | LAB_AST | 93.36±119.88 |
|  | 27 | Serum apolipoprotein AI, g/L | LAB_aPO A1 | 1.16±0.26 |
|  | 28 | Serum apolipoprotein B, g/L | LAB_aPO B | 0.80±0.22 |
|  | 29 | Serum direct bilirubin, umol/L | LAB_DBil | 4.69±2.57 |
|  | 30 | Serum total cholesterol, mmol/L | LAB_TC | 4.31±1.03 |
|  | 31 | Serum total bilirubin, umol/L | LAB_TBiL | 14.27±6.74 |
|  | 32 | Serum total bile acid, umol/L | LAB_TBA | 2.88±3.44 |
|  | 33 | Total serum protein, g/L | LAB_STP | 61.73±5.83 |
|  | 34 | International Normalized Ratio of Plasma, INR | LAB_INR | 1.04±0.13 |
|  | 35 | Plasma activated partial thromboplastin time, second | LAB_APTT | 39.04±11.21 |
|  | 36 | Plasma thrombin time in, second | LAB_TT | 18.70±17.85 |
|  | 37 | Plasma prothrombin time activity, % | LAB_PTA | 96.46±14.34 |
|  | 38 | Plasma prothrombin time, second | LAB_PT | 13.64±1.25 |
|  | 39 | Plasma fibrinogen, g/l | LAB_FIB | 3.83±1.13 |
|  | 40 | Plasma plasma D dimer, ug/ml | LAB_D-Dimer | 0.89±2.04 |
|  | 41 | Serum thyroid stimulating hormone, uIU/ml | LAB_TSH | 1.94±4.18 |
|  | 42 | Serum thyroxine, ug/dl | LAB_T4 | 7.88±2.04 |
| Radiological features | 43 | Normal left ventricular ejection fraction | RF_1 | 996(59.61%) |
|  | 44 | Left atrium and left ventricle enlarged | RF_2 | 131(7.84%) |
|  | 45 | The three valves of the aortic valve are well opened and closed | RF_3 | 800(47.88%) |
|  | 46 | The three valves of the aortic valve open well | RF_4 | 367(21.96%) |
|  | 47 | Three valves of the aortic valve have normal echo | RF_5 | 759(45.42%) |
|  | 48 | Echo enhancement of the three valves of the aortic valve | RF_6 | 419(25.07%) |
|  | 49 | Poor closure of the three valves of the aortic valve | RF_7 | 384(22.98%) |
|  | 50 | The inner diameter of the right atrium is normal | RF_8 | 248(14.84%) |
|  | 51 | Normal inner diameter of dual chamber outflow tract | RF_9 | 1283(76.78%) |
|  | 52 | The ventricular septum and the posterior wall of the left ventricle open well | RF_10 | 56(3.35%) |
|  | 53 | Normal echo of the ventricular septum and the posterior wall of the left | RF_11 | 64(3.83%) |
|  | 54 | Ventricular septum and left ventricular posterior wall thickness are normal | RF_12 | 834(49.91%) |
|  | 55 | The thickness of the ventricular septum and the posterior wall of the left | RF_13 | 149(8.92%) |
|  | 56 | Symmetrical thickening of the ventricular septum and the posterior wall of the left | RF_14 | 298(17.83%) |
|  | 57 | Poor closure of the ventricular septum and the posterior wall of the left | RF_15 | 65(3.89%) |
|  | 58 | Good tricuspid valve opening and closing | RF_16 | 612(36.62%) |
|  | 59 | The tricuspid valve opens well | RF_17 | 688(41.17%) |
|  | 60 | Normal tricuspid valve echo | RF_18 | 1300(77.80%) |
|  | 61 | Poor tricuspid valve closure | RF_19 | 688(41.17%) |
|  | 62 | The diameter of the remaining rooms is normal | RF_20 | 400(23.94%) |
|  | 63 | The room diameter is normal | RF_21 | 1011(60.50%) |
|  | 64 | Pulmonary valve opening and closing is good | RF_22 | 1161(69.48%) |
|  | 65 | Normal pulmonary valve echo | RF_23 | 1295(77.50%) |
|  | 66 | Good mitral valve opening and closing | RF_24 | 361(21.60%) |
|  | 67 | Normal mitral valve echo | RF_25 | 1120(67.03%) |
|  | 68 | The posterior leaflet of the mitral valve is well opened and closed | RF_26 | 14(0.84%) |
|  | 69 | Echo enhancement of posterior mitral valve leaflet | RF_27 | 64(3.83%) |
|  | 70 | Mediastinum shifts to the left with position | RF_28 | 1(0.06%) |
|  | 71 | No abnormality in the shape of the heart shadow | RF_29 | 760(45.48%) |
|  | 72 | Sharp costophrenic angle | RF_30 | 1154(69.06%) |
|  | 73 | Roughly symmetrical bilateral thorax | RF_31 | 1256(75.16%) |
|  | 74 | No widening of the mediastinum | RF_32 | 91(5.45%) |
|  | 75 | Mediastinal position is centered without displacement | RF_33 | 1235(73.91%) |
|  | 76 | Heart shadow enlargement | RF_34 | 173(10.35%) |
|  | 77 | Bilateral diaphragm smoothing | RF_35 | 1147(68.64%) |
|  | 78 | Costophrenic angle is not sharp | RF_36 | 37(2.21%) |
| Disease diagnoses | 79 | Ischemic heart disease | DIS_IHD | 1671(100.00%) |
|  | 80 | Hypertension | DIS_HYP | 992(59.37%) |
|  | 81 | Heart disease complications | DIS_HDC | 762(45.60%) |
|  | 82 | diabetes | DIS_DM | 612(36.62%) |
|  | 83 | Heart failure | DIS_HF | 914(54.70%) |
|  | 84 | Phlebitis and thrombophlebitis | DIS_PT | 180(10.77%) |
|  | 85 | Cerebrovascular disease | DIS_CD | 281(16.82%) |
|  | 86 | Metabolic disorders | DIS_MD | 365(21.84%) |
|  | 87 | Arrhythmia | DIS_Ar | 255(15.26%) |
|  | 88 | Other circulatory system diseases | DIS_OCSD | 410(24.54%) |
|  | 89 | Respiratory diseases | DIS_RD | 445(26.63%) |
|  | 90 | other illnesses | DIS_other | 529(31.66%) |
|  | 91 | Diseases of the genitourinary system | DIS_GS | 230(13.76%) |
|  | 92 | Digestive system diseases | DIS_DIG | 224(13.41%) |
|  | 93 | Other endocrine system diseases | DIS_OESD | 80(4.79%) |
| Procedures | 94 | Diagnostic ultrasound | PRO_DU | 1378(82.47%) |
|  | 95 | Contrast Agent Cardiovascular Angiography | PRO_CACA | 1127(67.44%) |
|  | 96 | Respiratory therapy | PRO_RT | 1113(66.61%) |
|  | 97 | Vascular operations such as stent implantation | PRO_VO | 912(54.58%) |
|  | 98 | Cardiovascular surgery like cardiopulmonary bypass | PRO_CS | 1099(65.77%) |
|  | 99 | Chest X-ray | PRO_X-ray | 847(50.69%) |
|  | 100 | Non-surgical diagnostic examination of the heart and blood vessels | PRO_NSDEHBV | 643(38.48%) |
|  | 101 | Other surgery | PRO_other | 652(39.02%) |
| Medications | 102 | ARB drugs | DRUG_ARB | 91(5.45%) |
|  | 103 | B blockers | DRUG_B | 518(31.00%) |
|  | 104 | Calcium channel blockers | DRUG_CCB | 130(7.78%) |
|  | 105 | Heparin drugs | DRUG_Hep | 589(35.25%) |
|  | 106 | Antiplatelet drugs | DRUG_Ant | 596(35.67%) |
|  | 107 | Statins | DRUG_Sta | 579(34.65%) |
|  | 108 | ACEI drugs | DRUG_ACEI | 519(31.06%) |
