# Supplementary material for: Improving the Performance of Outcome Prediction for Inpatients With Acute Myocardial Infarction Based on Embedding Representation Learned From Electronic Medical Records: Development and Validation Study
Source: J Med Internet Res. 2022 Aug 3;24(8):e37486. doi: 10.2196/37486 (PMC9386580; doi:10.2196/37486)
Supplement: Multimedia Appendix 2 [file jmir_v24i8e37486_app2.docx]

**Multimedia Appendix 2.** Patient features of samples in the public MIMIC-III data set.

| Feature categories | ID | Feature groups | Feature groups abbreviation | Mean±Standard deviation or N(%) |
| --- | --- | --- | --- | --- |
| Demographic features | 1 | Age, year | Age | 68.34±11.94 |
|  | 2 | Gender, male | Gender | 1855(61.63%) |
| Laboratory tests | 3 | Blood pH, units | LAB_BPH | 7.37±0.09 |
|  | 4 | PO2, mm Hg | LAB_PO2 | 191.49±130.88 |
|  | 5 | PCO2, mm Hg | LAB_PCO2 | 42.17±10.81 |
|  | 6 | Base Excess, mEq/L | LAB_BE | -0.78±4.64 |
|  | 7 | Calculated Total CO2, mEq/L | LAB_TCO2 | 25.25±4.86 |
|  | 8 | Creatine Kinase, IU/L | LAB_CK | 594.59±2282.33 |
|  | 9 | Creatine Kinase, MB Isoenzyme, ng/mL | LAB_CK-MB | 49.13±98.46 |
|  | 10 | Basophils, % | LAB_BR | 0.34±0.39 |
|  | 11 | Eosinophils, % | LAB_ER | 1.31±1.77 |
|  | 12 | Lymphocytes, % | LAB_LR | 14.81±9.06 |
|  | 13 | Monocytes, % | LAB_MR | 4.51±2.23 |
|  | 14 | Neutrophils, % | LAB_NR | 77.58±11.33 |
|  | 15 | Urine pH, units | LAB_UPH | 5.77±0.80 |
|  | 16 | Troponin T, ng/mL | LAB_TT | 1.36±2.82 |
|  | 17 | Bilirubin, Total, mg/dL | LAB_TBiL | 0.77±0.93 |
|  | 18 | Asparate Aminotransferase, IU/L | LAB_AST | 111.36±401.23 |
|  | 19 | Alanine Aminotransferase, IU/L | LAB_ALT | 62.02±187.66 |
|  | 20 | Alkaline Phosphatase, IU/L | LAB_ALP | 96.05±82.94 |
|  | 21 | Specific Gravity | LAB_SG | 1.02±0.01 |
| Radiological features | 22 | No pericardial effusion | RF1 | 1960(65.12%) |
|  | 23 | Mildly thickened mitral valve leaflets | RF2 | 1428(47.44%) |
|  | 24 | The mitral valve leaflets are mildly thickened | RF3 | 1380(45.85%) |
|  | 25 | Normal RV chamber size and free wall motion | RF4 | 1286(42.72%) |
|  | 26 | Normal tricuspid valve leaflets | RF5 | 1266(42.06%) |
|  | 27 | Mildly thickened aortic valve leaflets | RF6 | 1143(37.97%) |
|  | 28 | Normal LV wall thickness | RF7 | 1017(33.79%) |
|  | 29 | Regional LV wall motion abnormalities | RF8 | 1017(33.79%) |
|  | 30 | Normal pulmonic valve leaflet | RF9 | 903(30.00%) |
|  | 31 | Normal aortic diameter | RF10 | 935(31.06%) |
|  | 32 | The left atrium is mildly dilated | RF11 | 891(29.60%) |
|  | 33 | Normal RA size | RF12 | 928(30.83%) |
|  | 34 | Mild symmetric LVH | RF13 | 824(27.38%) |
|  | 35 | Normal LV cavity size | RF14 | 807(26.81%) |
|  | 36 | The aortic valve leaflets (3) are mildly thickened | RF15 | 628(20.86%) |
|  | 37 | Normal mitral valve leaflets | RF16 | 578(19.20%) |
|  | 38 | Mildly dilated RA | RF17 | 550(18.27%) |
|  | 39 | Normal aortic valve leaflets | RF18 | 534(17.74%) |
|  | 40 | Mildly thickened tricuspid valve leaflets | RF19 | 478(15.88%) |
|  | 41 | Right ventricular chamber size and free wall motion are normal | RF20 | 560(18.60%) |
|  | 42 | Normal LA size | RF21 | 563(18.70%) |
|  | 43 | Moderate cardiomegaly | RF22 | 336(11.16%) |
|  | 44 | Heart size is normal | RF23 | 323(10.73%) |
|  | 45 | mild cardiomegaly | RF24 | 380(12.62%) |
|  | 46 | The aortic root is normal in diameter | RF25 | 364(12.09%) |
|  | 47 | mediastinal contours are stable | RF26 | 315(10.47%) |
|  | 48 | The right atrium is normal in size | RF27 | 307(10.20%) |
|  | 49 | Overall normal LVEF | RF28 | 307(10.20%) |
|  | 50 | Heart is enlarged | RF29 | 219(7.28%) |
|  | 51 | Atheroma in the descending thoracic aorta | RF30 | 314(10.43%) |
|  | 52 | Aorta is tortuous | RF31 | 160(5.32%) |
|  | 53 | Pulmonary hypertension | RF32 | 182(6.05%) |
|  | 54 | Atheroma in the aortic arch | RF33 | 199(6.61%) |
|  | 55 | The tricuspid valve leaflets are normal | RF34 | 139(4.62%) |
| Disease diagnoses | 56 | Certain infectious and parasitic diseases | DIS_CIPD | 1766(58.67%) |
|  | 57 | Neoplasms | DIS_Neo | 372(12.36%) |
|  | 58 | Endocrine, nutritional and metabolic diseases | DIS_ENMD | 2469(82.03%) |
|  | 59 | Diseases of the blood and blood-forming organs and certain disorders involving the immune mechanism | DIS_BLOOD | 1380(45.85%) |
|  | 60 | Mental and behavioral disorders | DIS_MBD | 900(29.90%) |
|  | 61 | Diseases of the nervous system | DIS_NS | 973(32.33%) |
|  | 62 | Diseases of arteries, arterioles, and capillaries | DIS_PC | 670(22.26%) |
|  | 63 | Diseases of pulmonary circulation | DIS_AAC | 391(12.99%) |
|  | 64 | Hypertensive disease | DIS_HYP | 2230(74.09%) |
|  | 65 | Diseases of veins and lymphatics, and other diseases of circulatory system | DIS_OCSD | 694(23.06%) |
|  | 66 | Chronic rheumatic heart disease | DIS_CRHD | 183(6.08%) |
|  | 67 | Cerebrovascular disease | DIS_CRE | 452(15.02%) |
|  | 68 | Other forms of heart disease | DIS_OHD | 2348(78.01%) |
|  | 69 | Ischemic heart disease | DIS_IHD | 2905(96.51%) |
|  | 70 | Diseases of the respiratory system | DIS_RD | 1658(55.08%) |
|  | 71 | Diseases of the digestive system | DIS_DIG | 1260(41.86%) |
|  | 72 | Diseases of the genitourinary system | DIS_GS | 1658(55.08%) |
|  | 73 | Diseases of the skin and subcutaneous tissue | DIS_SST | 553(18.37%) |
|  | 74 | Congenital malformations, deformations and chromosomal abnormalities | DIS_CA | 105(3.49%) |
|  | 75 | Diseases of the musculoskeletal system and connective tissue | DIS_MSCT | 674(22.39%) |
|  | 76 | Symptoms, signs and abnormal clinical and laboratory findings, not elsewhere classified | DIS_SSACLF | 1324(43.99%) |
|  | 77 | Injury, poisoning and certain other consequences of external causes | DIS_IP | 1525(50.66%) |
|  | 78 | Other disease | DIS_other | 1876(62.33%) |
|  | 79 | Factors influencing health status and contact with health services | DIS_FIHS | 1070(35.55%) |
| Procedures | 80 | Operations on the nervous system | PRO_NS | 494(16.41%) |
|  | 81 | Operations on the endocrine system | PRO_ES | 446(14.82%) |
|  | 82 | Operations on valves and septa of heart | PRO_VSH | 177(5.88%) |
|  | 83 | Operations on vessels of heart | PRO_VH | 1577(52.39%) |
|  | 84 | Other operations on heart and pericardium | PRO_OOHP | 1718(57.08%) |
|  | 85 | Incision, excision, and occlusion of vessels | PRO_IEOV | 1072(35.61%) |
|  | 86 | Other operations on vessels | PRO_OOV | 1095(36.38%) |
|  | 87 | Operations on the respiratory system | PRO_RS | 318(10.56%) |
|  | 88 | Monitoring and evaluation(Procedure) | PRO_ME | 231(7.67%) |
|  | 89 | Physical therapy, respiratory therapy, rehabilitation, and related procedures | PRO_PRR | 112(3.72%) |
|  | 90 | Nonoperative intubation and irrigation | PRO_NII | 981(32.59%) |
|  | 91 | Operations on the digestive system | PRO_DS | 425(14.12%) |
|  | 92 | Diagnostic Radiology | PRO_DR | 1899(63.09%) |
|  | 93 | Replacement and removal of therapeutic appliances | PRO_RRDA | 125(4.15%) |
|  | 94 | Operations on the integumentary system | PRO_IS | 231(7.67%) |
|  | 95 | Operation on other parts | PRO_OP | 118(3.92%) |
|  | 96 | Other nonoperative procedures | PRO_ONP | 1472(48.90%) |
|  | 97 | Miscellaneous diagnostic and therapeutic procedures | PRO_MDTP | 664(22.06%) |
| Medications | 98 | ARB drugs | DRUG_ARB | 280(9.30%) |
|  | 99 | B blockers | DRUG_B | 2829(93.99%) |
|  | 100 | Calcium channel blockers | DRUG_CCB | 821(27.28%) |
|  | 101 | Heparin drugs | DRUG_Hep | 2699(89.67%) |
|  | 102 | Antiplatelet drugs | DRUG_Ant | 2776(92.23%) |
|  | 103 | Statins | DRUG_Sta | 2540(84.39%) |
|  | 104 | ACEI drugs | DRUG_ACEI | 1835(60.96%) |
