# Supplementary material for: Improving the Performance of Outcome Prediction for Inpatients With Acute Myocardial Infarction Based on Embedding Representation Learned From Electronic Medical Records: Development and Validation Study
Source: J Med Internet Res. 2022 Aug 3;24(8):e37486. doi: 10.2196/37486 (PMC9386580; doi:10.2196/37486)
Supplement: Multimedia Appendix 3 [file jmir_v24i8e37486_app3.docx]

**Multimedia Appendix 3.** Predictive performance of skip-gram–based embedding representations with different combinations of the size of the context window and the dimension of the embedding vector.

| Parameter settings | | Evaluation metrics (mean[95% confidence interval]) | | | | | |
| --- | --- | --- | --- | --- | --- | --- | --- |
| Embedding dimension | **Window size** | **AUC** | **AUCPR** | **F1-score** | **Precision** | **Recall** | **Accuracy** |
| 50 | **5** | 0.750 (0.711 ,0.789) | 0.144 (0.111 ,0.177) | 0.265 (0.208 ,0.322) | 0.160 (0.119 ,0.201) | 0.806 (0.700 ,0.912) | 0.635 (0.529 ,0.741) |
| 50 | **10** | 0.754 (0.713 ,0.795) | 0.147 (0.114 ,0.180) | 0.271 (0.216 ,0.326) | 0.164 (0.123 ,0.205) | 0.796 (0.690 ,0.902) | 0.651 (0.555 ,0.747) |
| 50 | **15** | 0.748 (0.705 ,0.791) | 0.141 (0.110 ,0.172) | 0.261 (0.206 ,0.316) | 0.157 (0.116 ,0.198) | 0.813 (0.690 ,0.936) | 0.624 (0.514 ,0.734) |
| 50 | **20** | 0.735 (0.694 ,0.776) | 0.139 (0.110 ,0.168) | 0.259 (0.210 ,0.308) | 0.156 (0.119 ,0.193) | 0.782 (0.662 ,0.902) | 0.635 (0.529 ,0.741) |
| 100 | **5** | 0.806 (0.767 ,0.845) | 0.172 (0.133 ,0.211) | 0.315 (0.256 ,0.374) | 0.199 (0.152 ,0.246) | 0.777 (0.677 ,0.877) | 0.725 (0.654 ,0.796) |
| 100 | **10** | 0.798 (0.759 ,0.837) | 0.166 (0.127 ,0.205) | 0.304 (0.243 ,0.365) | 0.189 (0.142 ,0.236) | 0.789 (0.695 ,0.883) | 0.706 (0.635 ,0.777) |
| 100 | **15** | 0.806 (0.767 ,0.845) | 0.167 (0.126 ,0.208) | 0.306 (0.239 ,0.373) | 0.191 (0.136 ,0.246) | 0.792 (0.682 ,0.902) | 0.706 (0.614 ,0.798) |
| 100 | **20** | 0.809 (0.770 ,0.848) | 0.174 (0.131 ,0.217) | 0.318 (0.251 ,0.385) | 0.201 (0.146 ,0.256) | 0.782 (0.676 ,0.888) | 0.726 (0.648 ,0.804) |
| 200 | **5** | 0.817 (0.780 ,0.854) | 0.175 (0.134 ,0.216) | 0.317 (0.254 ,0.380) | 0.199 (0.148 ,0.250) | 0.804 (0.698 ,0.910) | 0.717 (0.637 ,0.797) |
| 200 | **10** | 0.821 (0.782 ,0.860) | 0.177 (0.136 ,0.218) | 0.319 (0.256 ,0.382) | 0.199 (0.148 ,0.250) | 0.821 (0.711 ,0.931) | 0.714 (0.641 ,0.787) |
| 200 | **15** | 0.812 (0.773 ,0.851) | 0.171 (0.132 ,0.210) | 0.309 (0.246 ,0.372) | 0.192 (0.141 ,0.243) | 0.819 (0.709 ,0.929) | 0.702 (0.620 ,0.784) |
| 200 | **20** | 0.819 (0.782 ,0.856) | 0.174 (0.135 ,0.213) | 0.313 (0.254 ,0.372) | 0.195 (0.148 ,0.242) | 0.824 (0.730 ,0.918) | 0.706 (0.643 ,0.769) |
| 300 | **5** | 0.833 (0.796 ,0.870) | 0.183 (0.134 ,0.232) | 0.326 (0.252 ,0.400) | 0.205 (0.144 ,0.266) | 0.832 (0.736 ,0.928) | 0.719 (0.633 ,0.805) |
| 300 | **10** | **0.836 (0.799 ,0.873)** | **0.186 (0.133 ,0.239)** | **0.330 (0.248 ,0.412)** | **0.207 (0.134 ,0.280)** | **0.839 (0.714 ,0.964)** | **0.720 (0.593 ,0.847)** |
| 300 | **15** | 0.830 (0.793 ,0.867) | 0.181 (0.128 ,0.234) | 0.325 (0.241 ,0.409) | 0.206 (0.132 ,0.280) | 0.819 (0.696 ,0.942) | 0.718 (0.610 ,0.826) |
| 300 | **20** | 0.832 (0.795 ,0.869) | 0.182 (0.133 ,0.231) | 0.326 (0.252 ,0.400) | 0.205 (0.140 ,0.270) | 0.826 (0.716 ,0.936) | 0.718 (0.622 ,0.814) |

**Note**: We set the size of context window to 5, 10, 15 and 20, and the dimension of embedding vector to 50,100, 200 and 300. The feature embeddings were trained by the skip-gram algorithm using the random selection method to determine the context window, and the patient embedding representation were the average of feature embeddings. The patient embeddings were applied to build patient mortality prediction, which was evaluated by the area under the receiver operating characteristic curve (AUC), the area under the Precision Recall curve (AUCPR), F1-score, precision, recall and accuracy. The skip-gram model and the prediction model were conducted for 100 times, respectively, and the mean and its 95% confidence interval were reported. It was shown that the combination of window size of 10 and embedding dimension of 300 showed superior performance, achieving the highest AUC, AUCPR, F1-score and other evaluation metrics.
