# Supplementary material for: Improving the Performance of Outcome Prediction for Inpatients With Acute Myocardial Infarction Based on Embedding Representation Learned From Electronic Medical Records: Development and Validation Study
Source: J Med Internet Res. 2022 Aug 3;24(8):e37486. doi: 10.2196/37486 (PMC9386580; doi:10.2196/37486)
Supplement: Multimedia Appendix 4 [file jmir_v24i8e37486_app4.docx]

**Multimedia Appendix 4.** Predictive performance of patient representation methods on the public data set.

| Feature set and representation methods | | | AUROC^a^, mean (95% CI) | AUPRC^b^, mean (95% CI) | F1-score, mean (95% CI) |
| --- | --- | --- | --- | --- | --- |
| **Entire feature set** | | |  |  |  |
|  | **Embedding-based representation methods** | |  |  |  |
|  |  | CON_EM_WGT^c^ | 0.878 (0.847-0.909) | 0.220 (0.165-0.275) | 0.376 (0.296-0.456) |
|  |  | CON_EM_AVE^d^ | 0.850 (0.815-0.885) | 0.200 (0.149-0.251) | 0.352 (0.276-0.428) |
|  |  | ANT_EM_WGT^e^ | 0.872 (0.839-0.905) | 0.214 (0.163-0.265) | 0.368 (0.294-0.442) |
|  |  | ANT_EM_AVE^f^ | 0.834 (0.797-0.871) | 0.187 (0.140-0.234) | 0.332 (0.259-0.405) |
|  |  | RAN_EM_WGT^g^ | 0.863 (0.828-0.898) | 0.207 (0.148-0.266) | 0.359 (0.273-0.445) |
|  |  | RAN_EM_AVE^h^ | 0.834 (0.797-0.871) | 0.188 (0.135-0.241) | 0.334 (0.252-0.416) |
|  | **Reference representation methods** | |  |  |  |
|  |  | DIS_AE^i^ | 0.799 (0.768-0.830) | 0.167 (0.134-0.200) | 0.304 (0.255-0.353) |
|  |  | DIS_FS^j^ | 0.836 (0.803-0.869) | 0.187 (0.144-0.230) | 0.330 (0.261-0.399) |
|  |  | Discretization | 0.847 (0.814-0.880) | 0.196 (0.145-0.247) | 0.344 (0.266-0.422) |
|  |  | Mixture | 0.841 (0.806-0.876) | 0.188 (0.143-0.233) | 0.333 (0.259-0.407) |
| **Treatment-free feature set** | | |  |  |  |
|  | **Embedding-based representation methods** | |  |  |  |
|  |  | CON_EM_WGT | 0.784 (0.737-0.831) | 0.163 (0.118-0.208) | 0.302 (0.224-0.380) |
|  |  | CON_EM_AVE | 0.796 (0.751-0.841) | 0.161 (0.118-0.204) | 0.295 (0.222-0.368) |
|  |  | ANT_EM_WGT | 0.773 (0.724-0.822) | 0.157 (0.112-0.202) | 0.294 (0.214-0.374) |
|  |  | ANT_EM_AVE | 0.784 (0.739-0.829) | 0.155 (0.116-0.194) | 0.286 (0.217-0.355) |
|  |  | RAN_EM_WGT | 0.764 (0.715-0.813) | 0.154 (0.113-0.195) | 0.289 (0.215-0.363) |
|  |  | RAN_EM_AVE | 0.772 (0.727-0.817) | 0.146 (0.109-0.183) | 0.269 (0.208-0.330) |
|  | **Reference representation methods** | |  |  |  |
|  |  | DIS_AE | 0.683 (0.644-0.722) | 0.120 (0.095-0.145) | 0.229 (0.186-0.272) |
|  |  | DIS_FS | 0.703 (0.656-0.750) | 0.122 (0.093-0.151) | 0.232 (0.177-0.287) |
|  |  | Discretization | 0.722 (0.675-0.769) | 0.129 (0.096-0.162) | 0.245 (0.182-0.308) |
|  |  | Mixture | 0.736 (0.693-0.779) | 0.136 (0.103-0.169) | 0.255 (0.196-0.314) |

^a^AUROC: area under the receiver operating characteristic curve.

^b^AUPRC: area under the precision-recall curve.

^c^CON_EM_WGT: weighted sum of the consequent-based embedding representation.

^d^CON_EM_AVE: average of the consequent-based embedding representation.

^e^ANT_EM_WGT: weighted sum of the antecedent-based embedding representation.

^f^ANT_EM_AVE: average of the antecedent-based embedding representation.

^g^RAN_EM_WGT: weighted sum of the random selection–based embedding representation.

^h^RAN_EM_AVE: average of the random selection–based embedding representation.

^i^DIS_AE: discretization representations with features selection.

^j^DIS_FS: hidden vector of an autoencoder-based representation.
