# Supplementary material for: Improving the Performance of Outcome Prediction for Inpatients With Acute Myocardial Infarction Based on Embedding Representation Learned From Electronic Medical Records: Development and Validation Study
Source: J Med Internet Res. 2022 Aug 3;24(8):e37486. doi: 10.2196/37486 (PMC9386580; doi:10.2196/37486)
Supplement: Multimedia Appendix 5 [file jmir_v24i8e37486_app5.docx]

**Multimedia Appendix 5.** The average predictive performance of patient representation methods on the public and private data sets with and without treatment feature sets. Consistently superior predictive performance was achieved on both datasets with the entire feature set compared with the treatment-free feature set.
