# Supplementary material for: Improving the Performance of Outcome Prediction for Inpatients With Acute Myocardial Infarction Based on Embedding Representation Learned From Electronic Medical Records: Development and Validation Study
Source: J Med Internet Res. 2022 Aug 3;24(8):e37486. doi: 10.2196/37486 (PMC9386580; doi:10.2196/37486)
Supplement: Multimedia Appendix 6 [file jmir_v24i8e37486_app6.docx]

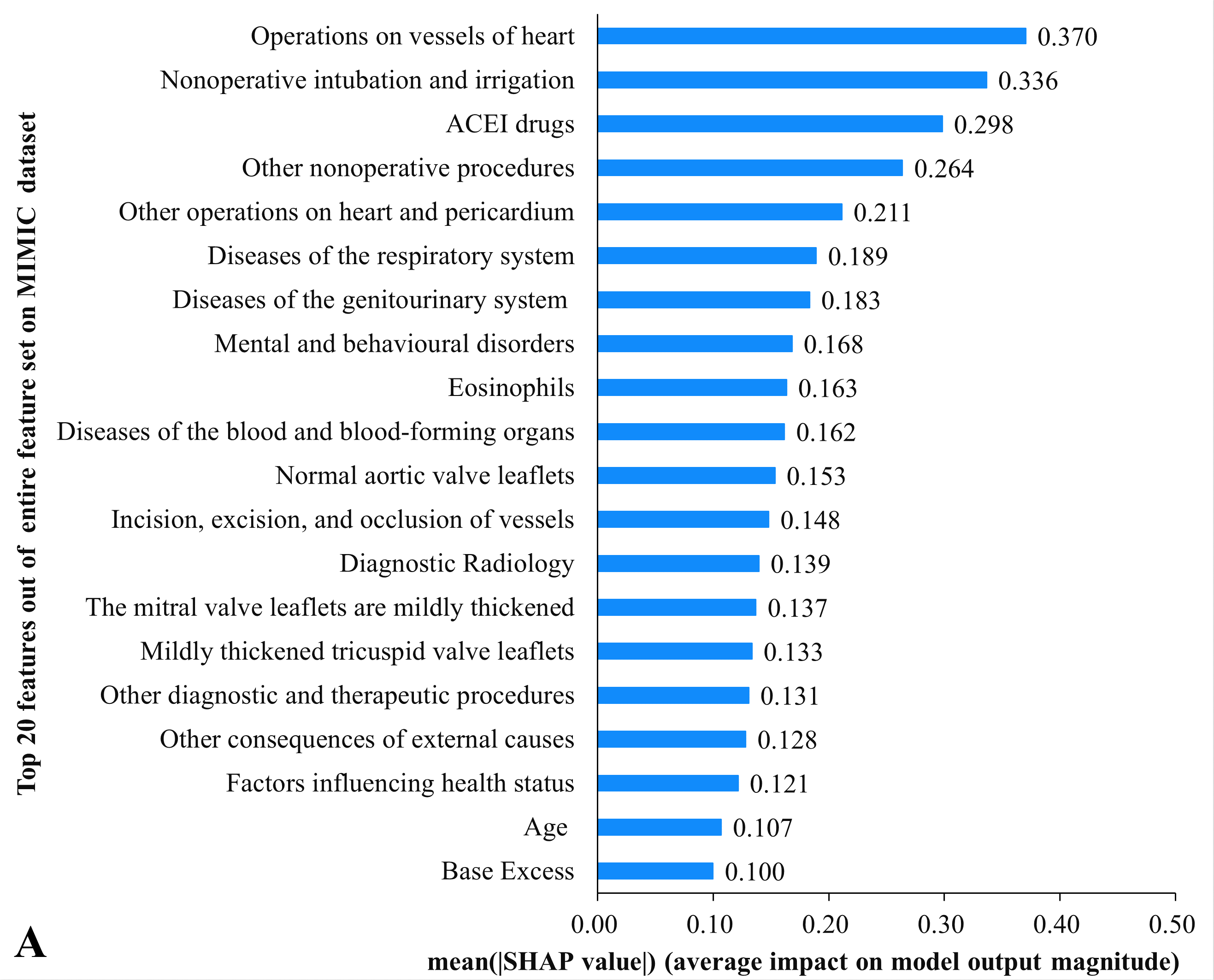

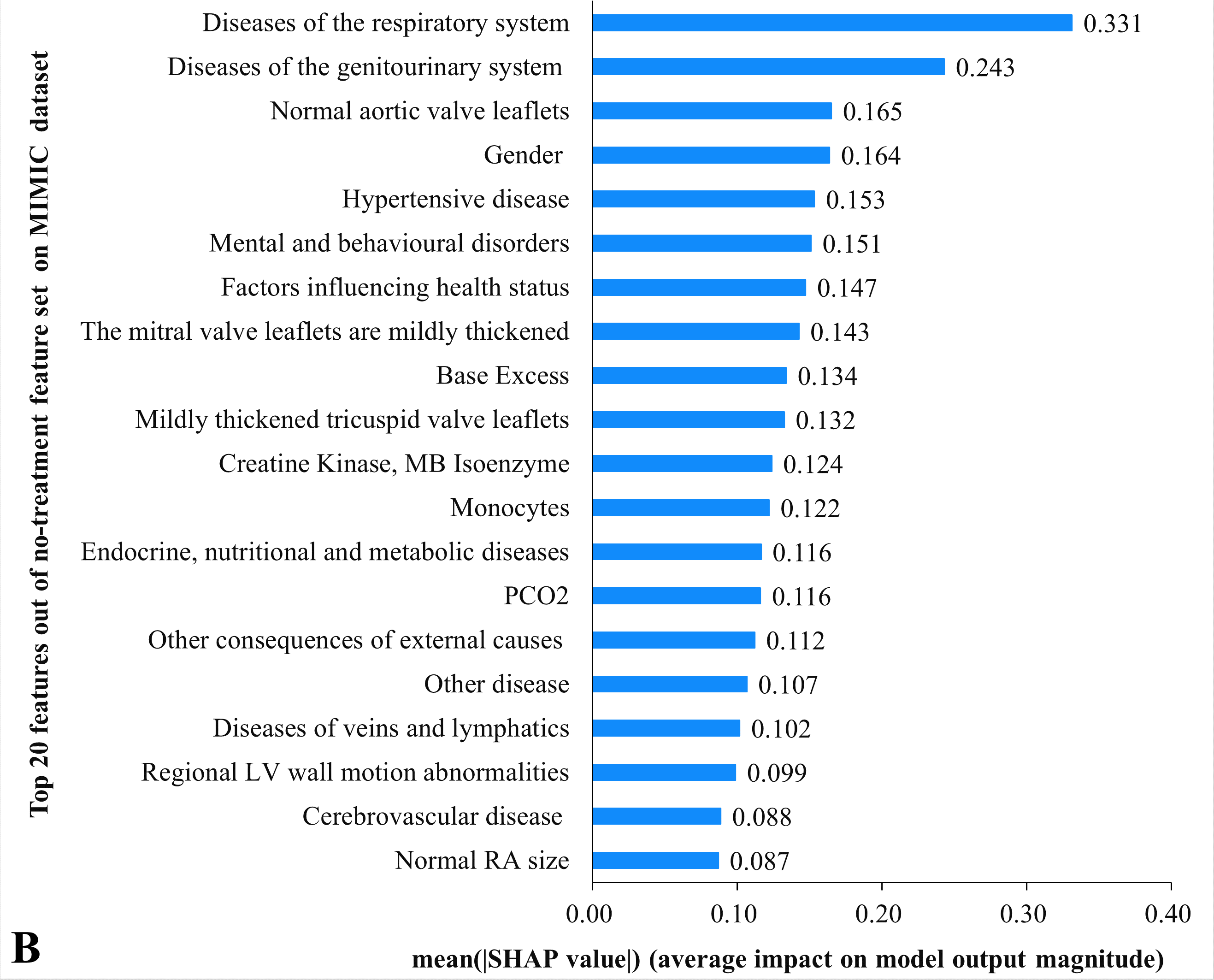


**Multimedia Appendix 6.** The mean absolute Shapley additive explanations (SHAP) values of the top 20 features of the public data set within the entire feature set (A) and the treatment-free feature set (B).
