# Supplementary material for: Improving the Performance of Outcome Prediction for Inpatients With Acute Myocardial Infarction Based on Embedding Representation Learned From Electronic Medical Records: Development and Validation Study
Source: J Med Internet Res. 2022 Aug 3;24(8):e37486. doi: 10.2196/37486 (PMC9386580; doi:10.2196/37486)
Supplement: Multimedia Appendix 7 [file jmir_v24i8e37486_app7.docx]

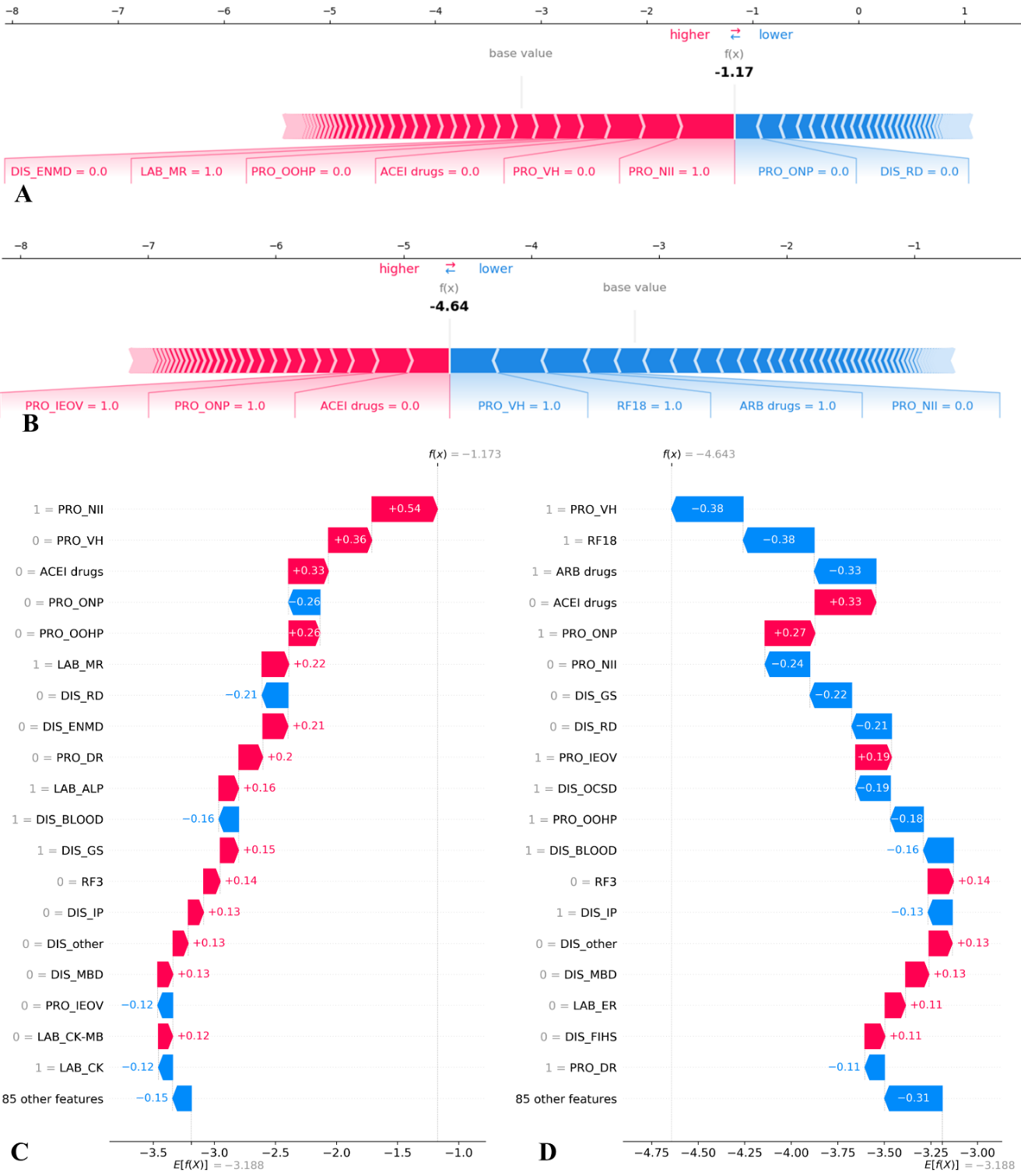


**Multimedia Appendix 7.** Shapley additive explanations (SHAP) values for a patient who died during hospital stay (A and C) and another patient who did not die (B and D) from the public data set with the entire feature set. A and B, all features with their SHAP values, and C and D, 20 features with the greatest absolute SHAP values. Features in blue tend to reduce the possibility of a patient being classified as positive (death in this study), while features in red do the contrary. Meaning of each abbreviated feature can be found in Multimedia Appendix 2.
